# Identifying escaped farmed salmon from fish scales using deep learning

**DOI:** 10.1093/biomethods/bpaf078

**Published:** 2025-11-26

**Authors:** Malte Willmes, Anders Varmann Aamodt, Børge Solli Andreassen, Lina Victoria Tuddenham Haug, Enghild Steinkjer, Gunnel M Østborg, Gitte Løkeberg, Peder Fiske, Geir R Brandt, Terje Mikalsen, Arne Siversten, Magnus Moustache, June Larsen Ydsti, Bjørn Florø-Larsen

**Affiliations:** Norwegian Institute for Nature Research, PO Box 5685, 7485 Torgarden, Trondheim, Norway; Simula Research Laboratory, Department of Applied AI, Kristian Augusts gate 23, 0164 Oslo, Norway; Simula Research Laboratory, Department of Applied AI, Kristian Augusts gate 23, 0164 Oslo, Norway; The Norwegian Environment Agency, P.O. Box 5672 Torgarden, N-7485 Trondheim, Norway; Norwegian Institute for Nature Research, PO Box 5685, 7485 Torgarden, Trondheim, Norway; Norwegian Institute for Nature Research, PO Box 5685, 7485 Torgarden, Trondheim, Norway; Norwegian Institute for Nature Research, PO Box 5685, 7485 Torgarden, Trondheim, Norway; Norwegian Institute for Nature Research, PO Box 5685, 7485 Torgarden, Trondheim, Norway; JBT Marel, Vestbygata 55B, 2003 Lillestrøm, Skedsmo, Norway; JBT Marel, Vestbygata 55B, 2003 Lillestrøm, Skedsmo, Norway; The Norwegian Environment Agency, P.O. Box 5672 Torgarden, N-7485 Trondheim, Norway; The Norwegian Environment Agency, P.O. Box 5672 Torgarden, N-7485 Trondheim, Norway; The Norwegian Environment Agency, P.O. Box 5672 Torgarden, N-7485 Trondheim, Norway; Norwegian Veterinary Institute, PO Box 4024 Angelltrøa, 7457 Trondheim, Norway

**Keywords:** fish scales, convolutional neural network, farmed salmon, escaped salmon

## Abstract

Escaped farmed salmon are a major concern for wild Atlantic salmon (*Salmo salar*) stocks in Norway. Fish scale analysis is a well-established method for distinguishing farmed from wild fish, but the process is labor and time intensive. Deep learning has recently been shown to automate this task with high accuracy, though typically on relatively small and geographically limited datasets. Here we train and validate a new convolutional neural network on nearly 90 000 scale images from two national archives, encompassing heterogeneous imaging protocols, hundreds of rivers, and time series extending back to the 1930s. The model achieved an F1 score of 0.95 on a large, independent test set, with predictions closely matching both genetic reference samples and known farmed-origin scales. By developing and testing this new model on a large and diverse dataset, we demonstrate that deep learning generalizes robustly across ecological and methodological contexts, supporting its use as a validated, large-scale tool for monitoring escaped farmed salmon.

## Introduction

The Atlantic salmon (*Salmo salar*) is an anadromous fish species native to the Atlantic Ocean and the rivers that drain into it and is of high ecological, cultural, and economic importance [1]. Over 2000 genetically distinct populations occur throughout its native range, however its abundance has declined over the past decades [2]. A number of different factors are thought to contribute to this decline, including habitat degradation, migration barriers, overexploitation, and the effects of introgression and increased disease and parasite load from escaped farmed salmon [1, 3].

Norway represents a critical region for Atlantic salmon because it is home to some of the largest remaining wild populations and also is one of the largest producers of farmed salmon [1, 4]. Consequently, protecting Atlantic salmon populations in Norway is an important management and conservation goal. Atlantic salmon abundance in Norway has declined by over 50% since the 1980s and is now at historically low levels [4]. The effects of escaped farmed salmon are thought to be one of the important drivers behind this decline. Norway produces over 1.5 million metric tons of farmed Atlantic salmon annually, predominantly in open-net pens situated along the coastline. Each year, approximately 200 000–300 000 farmed salmon escape into the wild due to infrastructure failures, human error, or extreme weather events [5].

These escaped salmon represent a substantial ecological and genetic threat to wild populations. Farmed salmon differ genetically from wild populations, having undergone selection for traits favorable in aquaculture, such as rapid growth and tolerance to crowded conditions [6]. Interbreeding between escaped farmed salmon and wild salmon leads to genetic introgression [7], causing reduced fitness and reduced adaptability in wild populations. Indeed, genetic analyses have shown that approximately two-thirds of wild salmon stocks in Norway carry genetic signatures indicative of farmed salmon interbreeding [5, 8]. Additionally, escaped salmon increase competition for limited resources, such as food and spawning habitats, potentially displacing wild salmon or reducing their reproductive success [9, 10]. Farmed salmon may also introduce pathogens and parasites such as sea lice (*Lepeophtheirus salmonis*), exacerbating pressures on wild salmon populations already vulnerable due to climate change and habitat degradation [1, 11]. To mitigate ecological and genetic risks of farmed salmon, improved containment measures, transitioning toward closed systems, and rigorous monitoring of escape events are needed.

Methods used to monitor escaped farmed salmon include genetic analyses, morphological assessments, and scale analysis [12]. Genetic analyses typically involve molecular markers to differentiate farmed from wild salmon populations, and enable precise identification of hybridization events [7, 8]. Morphological assessments examine physical characteristics such as body shape, fin erosion, and condition factors indicative of aquaculture conditions [9, 10], while scale analysis makes use of distinct growth patterns on fish scales to distinguish wild origin from farmed fish [12, 13]. Salmon scales grow by forming concentric rings on their surface, with the number and spacing of these rings corresponding to the somatic growth of the fish. Scales have been used to reconstruct somatic growth and life histories in salmon since the early 1900s [14], and are a commonly collected tissue to monitor Atlantic salmon [15, 16].

The morphological differences between scales of farmed and wild Atlantic salmon arise from fundamental contrasts in growth dynamics, environmental variability, and life-history. Farmed salmon are reared under highly controlled conditions, characterized by continuous feeding, stable temperatures, and reduced energetic costs due to confinement in cages. These conditions promote rapid and steady somatic growth, resulting in regularly spaced circuli with weak or absent seasonal checks [13]. In contrast, wild salmon experience pronounced seasonal and ontogenetic variation in growth driven by fluctuating temperature, prey availability, and migration between freshwater and marine habitats. This leads to irregular circuli spacing, distinct freshwater annuli, and abrupt transition zones marking the smolt migration and first marine growth [12, 13]. In aquaculture, mechanical abrasion and crowding may further modify the outer surface, producing regenerated or partially eroded scales. These patterns provide the biological foundation for visual classification by experts and define the textural and structural cues that convolutional networks exploit during automated recognition.

Beyond individual growth history, geographic and environmental variability further influence scale morphology in Atlantic salmon. Water temperature, productivity, photoperiod, and migration distance vary substantially among Norwegian river systems, producing local differences in growth rates and in the appearance of annuli and circuli spacing [1, 10]. For instance, northern populations with shorter growing seasons often show narrower circuli spacing and more prominent winter checks than southern stocks, while populations in warmer or more productive rivers exhibit more continuous growth patterns [1, 5, 10, 13]. These gradients introduce natural phenotypic variability that need to be taken into account when using scales to identify escaped farmed salmon.

The manual reading of scales is a labor-intensive process and often cost-prohibitive, making this an ideal opportunity for automated methods. The advancement of new semi-automated approaches [17] and deep learning models [18, 19] has the potential to fundamentally change how fish scales are analyzed. Current deep learning models can provide accuracy and precision comparable to manual scale reading, while being more reproducible and increasing information yield [18, 19]. However, most applications to date have relied on relatively limited datasets, leaving open the question of whether such models generalize across the spatial, temporal, and methodological diversity of national monitoring programs. To address this, we trained a new convolutional neural network (CNN) on nearly 90 000 Atlantic salmon scale images from the Norwegian Veterinary Institute (VI) and the Norwegian Institute for Nature Research (NINA). We established a standardized processing pipeline and evaluated the model against human scale readers and known-origin fish, providing a large-scale test of deep learning as a tool for classifying farmed versus wild salmon within Norway’s surveillance program.

## Materials and methods

### Data sources

**Figure 1:**
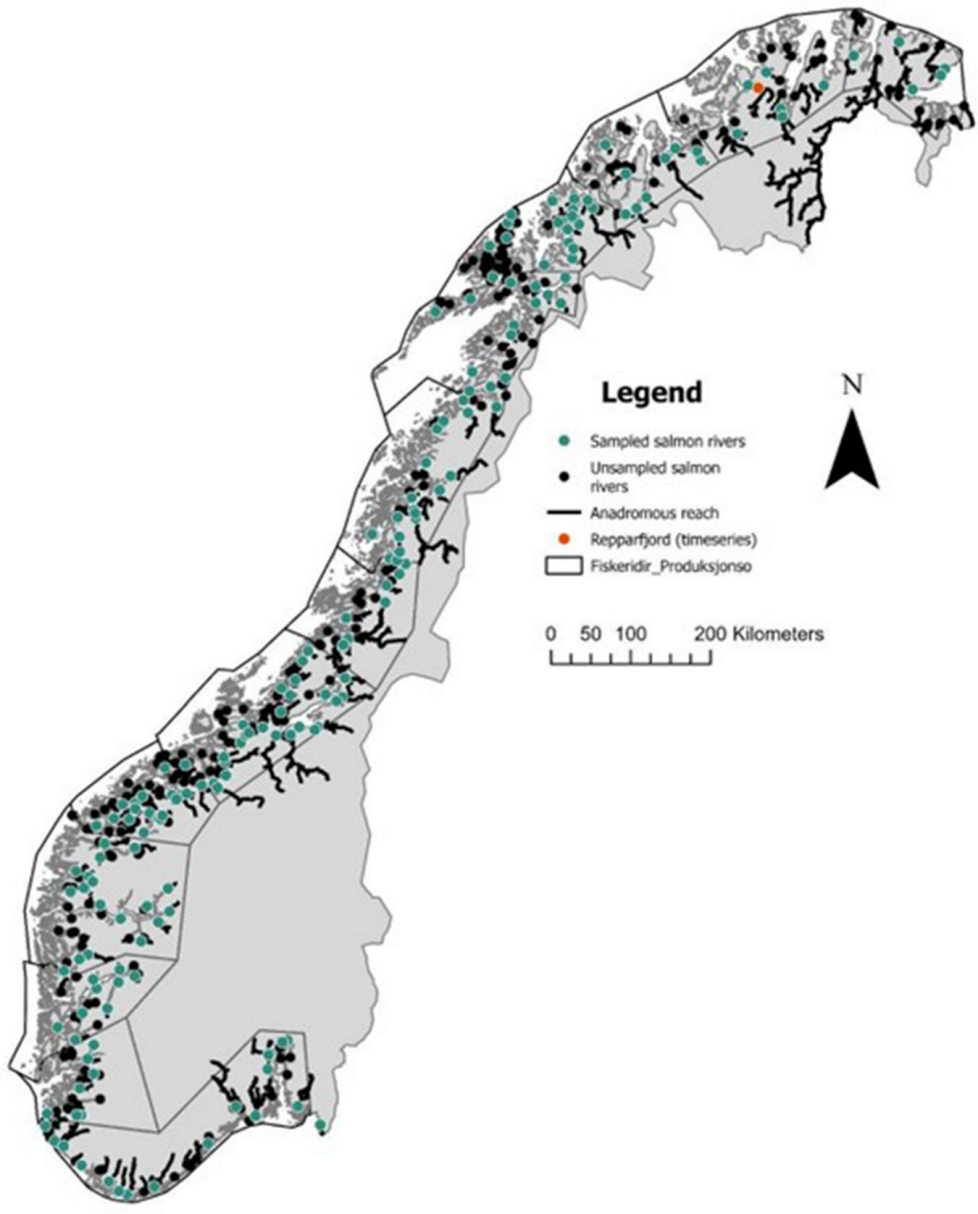
Map of Norway with the location of the Atlantic salmon watercourses, indicated by dots at the location of the river outlet. Green dots indicate a watercourse that was included in the scale samples, while black dots indicate an unsampled watercourse. Repparfjord, which is the location of the long time series data, is shown as an orange dot. Polygons represent salmon production zones. Data “Anadrome laksefisk” from Miljødirektoratet (https://kartkatalog.miljodirektoratet.no) and “Produksjonsområder” from Fiskeridirektoratet (https://open-data-fiskeridirektoratet-fiskeridir.hub.arcgis.com)

**Figure 2:**
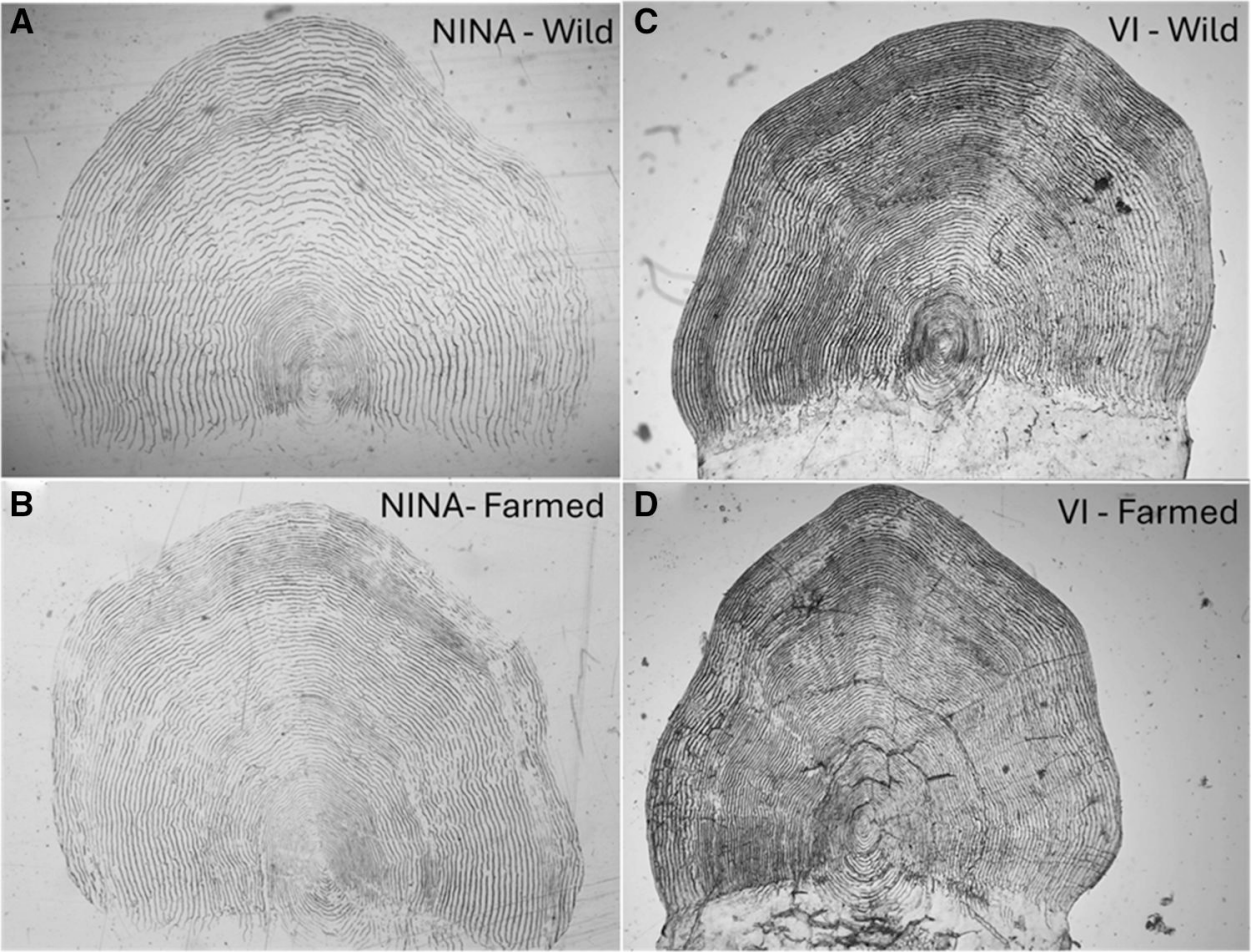
Scale image examples. NINA scale images of (A) wild, and (B) farmed salmon are taken from imprints, while VI scale images of (C) wild, and (D) farmed salmon are taken from the actual scales

Fish scale images were sourced from the Norwegian Veterinary Institute (VI) and from the Norwegian Institute for Nature Research (NINA). These samples cover a large temporal and spatial range with the aim to encompass among stock variability (Fig. 1, Table 1). The VI works directly on images of scales, while at NINA the protocol is to first create an imprint by pressing the scales onto a cellulose acetate slide with a roller and then imaging the resulting imprint (Fig. 2). As images were collected by many different research projects and across time, the magnification, resolution, and general quality of the original images are variable. Data cleaning removed a small percentage of the sourced images (3.4%, *n* = 3044), commonly images that were over-exposed (completely white or black) or that contained multiple, sometimes overlapping fish scales, or other image artifacts.

**Table 1.** Summary of fish scale image dataset by origin class and data split.

Class	Training set	Validation set	Test set	Sum
Wild	55 795	13 898	12 264	81 957
Farmed	5166	1313	1162	7641
Total images	60 961	15 211	13 426	89 598

The total dataset consisted of approximately 82 thousand images from VI and 7.5 thousand from NINA (**Table 1**) and covers hundreds of rivers across Norway and goes back to the early 1930s. The dataset is heavily class-imbalanced, with farmed salmon comprising approximately 8.5% of the total images compared to wild salmon. For the purpose of this model we classified hatchery reared salmon (*n* = 371) into the same category as farmed salmon, because they comprised only a tiny fraction of our available data. To train and evaluate the model performance, we randomly split the data into training, validation, and test set, approximately preserving the original class distribution. Of the overall dataset, 85% was allocated to training and validation, with an 80/20 split between them. The remaining 15% of the overall dataset was held out for testing.

### Image processing

The raw images in the dataset showed considerable variation in size and resolution. To standardize them and simplify processing, we first resized all images to fit within [720, 480] pixels, using Lanczos interpolation and scaling based on the longest side. Before being fed into the model, the images were further resized to [384, 512] and normalized using the standard ImageNet per-channel means of [0.485, 0.456, 0.406] and standard deviations of [0.229, 0.224, 0.225]. These steps were chosen to match the model’s required input dimensions and to ensure compatibility with the model’s pre-training weights. A range of augmentation steps were tested during the experimentation phase. The best results were obtained by incorporating additional augmentations during the training phase. Specifically, the images were converted to grayscale and then augmented using the AutoAugment strategy proposed by Cubuk *et al*. [20], applying the ImageNet policy, between the resizing and normalization.

### Deep learning model architecture and optimization

We selected the ResNeXt model architecture for its strong performance on image classification tasks [21]. Specifically, we used the ResNeXt-50 32x4d configuration with pretrained IMAGENET1K_V2 weights as the base model for transfer learning. To adapt the model for our task, we replaced its final fully-connected layer with a new one that outputs two classes. To preserve the benefits of the pretrained weights and support efficient convergence, we applied the OneCycleLR learning rate scheduler, which starts with a low learning rate. We configured it with a maximum learning rate of 6e-4, an initial division factor of 1.25, and a final division factor of 40. We used the AdamW optimizer with a weight decay of 1e-3 [22].

To address class imbalance, we used a weighted random sampler with class weights inversely proportional to class frequencies and set the epoch size to twice the number of samples in the minority class. This ensured a more balanced contribution from both classes during training, while the data augmentation described above added variability to the samples. Additionally, we employed focal loss (γ = 2, α = 0.5), which emphasizes harder examples and down-weights easier ones, making it well-suited for imbalanced classification tasks [23].

During the experimentation phase, we tested a range of hyperparameters before settling on the final training configuration. We also conducted ablations with different model architectures, including ConvNeXt [24] and visual transformer variants [25]. Due to computational constraints, model training during these tests was limited to 15–30 epochs, and performance was evaluated on the validation set. Based on these results, we conducted a longer training run of 150 epochs with a batch size of 64 to produce the model used for evaluation.

Model training and evaluation were conducted on NVIDIA GPUs using PyTorch (v2.2.2), torchvision (v0.17.2), and the Lightning framework (v2.2.1), which provided convenient access to model architectures, pretrained weights, and utilities such as data transforms.

### Model evaluation

The model was evaluated using the F1 score, which is more appropriate for imbalanced datasets compared to metrics like accuracy. Given that our dataset consisted of over 10x more “wild” classifications than “farmed,” accuracy alone would be misleading, as it could be artificially high simply by favoring the majority class. The F1 score, by combining both precision and recall, provides a more balanced assessment of the model’s performance, particularly in capturing how well the minority class is predicted.

## Results

**Figure 3:**
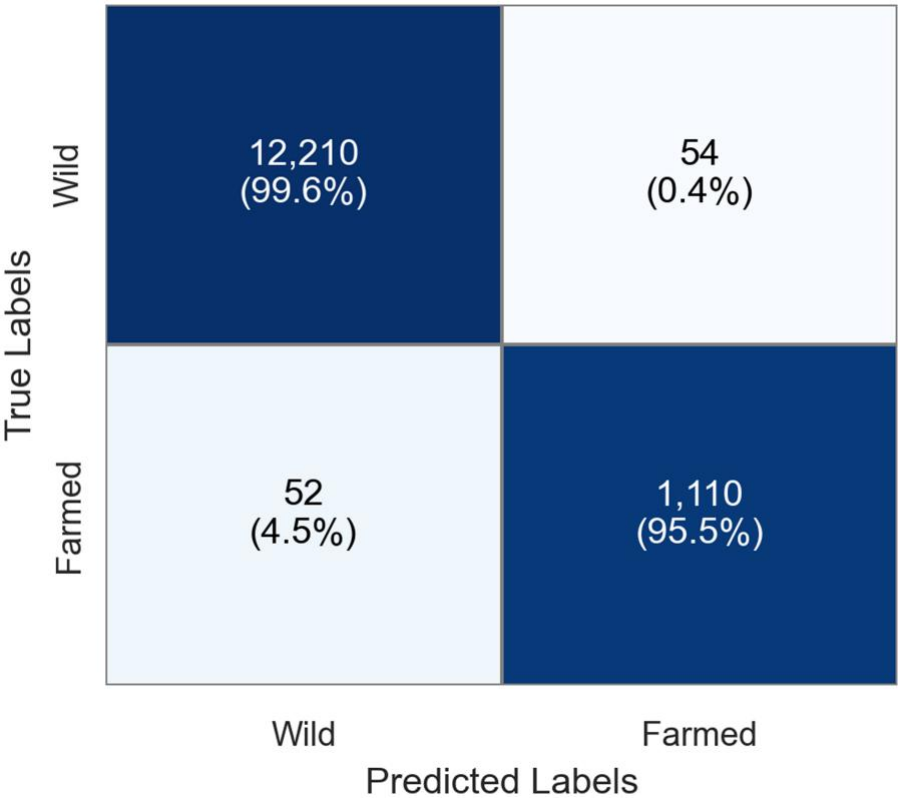
Confusion matrix for the classification model. Each cell displays the total number of classifications, with the row-normalized percentage shown in parentheses. The percentage indicates the proportion of samples from a true class (row) that were assigned to each predicted class (column)

After optimizing the model using the training and validation data, we selected the best performing checkpoint for testing. To ensure valid and unbiased results, we evaluated the final model on the separate, held out test-set that had been labeled by human scale readers and had not been used during training or validation. This approach was intended to mimic the conditions of a production environment and provide a realistic assessment of the model’s generalization performance. The model achieved excellent classification results with an F1 score of 0.95 on the unseen test-set (Table 2). The confusion matrices summarize our results over the independent test-set (Fig. 3).

To further investigate the model performance, we also evaluated the predicted labels against known origin samples from the test-set. For wild fish we made use of available genetic data that had provided a high probability (>71%) of wild origin. Of these genetically known to be wild fish from the test-set (*n* = 1890) the model correctly classified 99.9% (1 single mismatch). We could not apply the same test to fish genetically classified as farmed origin as they could simply represent wild offspring of a farmed lineage fish. To provide an assessment of farmed fish model performance we used fish from the test-set taken directly at fish farms (*n* = 438). For these fish the model classified 99.3% correctly (3 mismatches). These two metrics decouple the evaluation of the model performance from the human scale reader labels and thus provide an independent assessment.

**Table 2.** Summary of the model classification statistics on the test set (*n* = 13 426 scales).

Metric	Score
F1	0.95
Accuracy	0.99
Precision	0.95
Recall	0.96

## Discussion

Escaped farmed Atlantic salmon represent a significant threat to the genetic integrity and ecological sustainability of wild salmon populations in Norway [1, 5, 8]. To assess the impact of aquaculture on wild salmon populations, Norwegian authorities have established a comprehensive surveillance program in collaboration with eight research institutions and industry stakeholders. As part of this initiative, salmon scales are sampled annually from around 200 rivers throughout Norway [26] and analyzed by experts to retrieve a range of different life history metrics, including farmed vs. wild origin. The manual analysis of these scales is labor and time intensive, and often leads to a lag in the reporting of results back to the Norwegian authorities, which can delay management responses.

Here we developed a deep learning classification model to distinguish farmed from wild Atlantic salmon using scale images. The data pipeline and model can rapidly process images and provide predictions with associated confidence estimates. The model performed exceptionally well, achieving an F1 score of 95% in differentiating farmed and wild salmon across most salmon rivers in Norway from 2009 to 2023. Longer-term time series data were available from Repparfjorden (1932–2024), providing additional temporal depth to our analyses.

A similar model approach was taken by Vabø *et al*. [18], who used approximately 9000 scale images collected in Norway by the Institute of Marine Research in Bergen (2015–2018) and Rådgivende Biologer (2016–2017) to develop a CNN model predicting farmed vs. wild origin, spawning history, river age, and sea age. Their model achieved 97% accuracy in farmed vs. wild classification and accurately predicted spawning history and sea age, though river age predictions were less accurate [18]. In contrast, our study focuses exclusively on predicting fish origin but leverages a substantially larger and more geographically and temporally diverse dataset, covering hundreds of rivers across Norway. This broader dataset allowed us to test the applicability of the model across a wide range of ecological and temporal contexts, accounting for potential variation in scale growth patterns among rivers and over time.

Uncertainties in the assignments of farmed vs. wild origin among human scale readers are highly dependent on scale and image quality and on the time since escape, but accuracy of trained scale readers is generally assumed to be high ∼90% [12]. To independently evaluate model performance, we compared predictions against reference samples with known origin. Among genetically confirmed wild fish, the model classified 99.9% correctly. For farmed fish, we used samples taken directly from fish farms and found 99.3% accuracy. These results provide a robust validation independent of human scale-reader labels.

Despite its strong performance, the current model has limitations. It is specifically trained on Atlantic salmon scale images for origin classification and should not be applied to other species or to retrieve other life-history traits without further training. Variability in scale quality, image resolution, and sampling coverage could introduce biases, and environmental or ecological conditions not included in the sampled populations might affect scale growth patterns in ways not captured by the model. Careful supervision of confidence estimates, ongoing monitoring by human experts, and validation against independent confirmed-origin samples are essential to ensure that model outputs support conservation and management. Such supervision would involve flagging images with low confidence estimates for expert review, providing a necessary safeguard for ambiguous cases. This same mechanism could also serve to filter out potential “out-of-family” inputs, e.g. scales from other species, which may also fail to generate a high-confidence prediction. Finally, transparent reporting of model performance, uncertainties, and potential biases will be critical as these tools are integrated into long-term ecological monitoring.

While our proposed method benefits from the feature extraction capabilities of deep learning models, this advantage comes with a trade-off compared to some classical methods. Deep learning models are inherently black-boxes, i.e., they offer limited explainability of their predictions. In contrast, classical methods often provide more direct interpretability of the features driving their predictions. However, they typically require manually designed feature extractors to transform image data into model inputs. For heterogeneous datasets, like the one used in this study, there are significant variations in both scale morphology and image quality, making it difficult to define a single, robust set of features for manual extraction. Interpretability and explainability remain important, and future work could explore applying explainable AI (XAI) techniques to the current model and investigating hybrid methods inspired by classical approaches.

An additional important limitation of both manual and automated scale interpretation arises from cases where farmed salmon escape at a very early stage and subsequently grow under natural conditions. In such individuals, early circuli may reflect the uniform, rapid growth characteristic of hatchery rearing, whereas later rings show the irregular spacing typical of wild fish [12, 13]. This produces a mixed scale pattern that can challenge both human readers and automated classifiers. While the present model classifies scales based on the predominant morphology, it does not specifically identify early escapees from other farmed salmon. Future work could improve on this by adding one or more additional categories that account for early escaped farmed salmon with their mixed scale pattern.

The challenges posed by escaped farmed salmon are not unique to Norway. Countries such as the United Kingdom, Iceland, Canada, Chile, and the United States also face risks of genetic introgression, ecological disruption, and management delays associated with aquaculture escapees [27, 28]. The combination of systematic sampling, high quality image data, and Deep learning approaches provides a scalable framework that can be adapted across regions and species. This approach could support international efforts to safeguard wild salmon populations, inform regulatory decisions, and enhance the efficiency of long-term ecological surveillance. Beyond salmon, similar methodologies could be applied to other economically or ecologically important fish species, and other fish tissues with sequential growth chronologies such as otoliths [29, 30].

In addition to ecological monitoring, this framework could also support traceability in seafood markets by verifying whether salmon labeled as “wild-caught” show scale characteristics consistent with natural growth. Given the increasing interest in sustainable certification and origin verification, automated image-based classification may complement existing genetic or documentation-based traceability systems to ensure product authenticity.

The model developed in this study, and the model developed by Vabø *et al*. [18] provide excellent tools to aid in the rapid identification of escaped farmed salmon in Norway. The confidence estimates provided by these models can be used as a screening tool, removing all samples with high confidence in assignments, and flagging images with lower confidence estimates for additional human oversight or additional sample taking. This can greatly increase the management response time in cases of farmed salmon escapes. Looking forward, these models are poised to enhance the Norwegian Environment Agency’s capacity to safeguard wild salmon populations and to expand comprehensive life-cycle data for Atlantic salmon across Norway, supporting both conservation and management objectives.

## Data Availability

The authors are open to exploring data collaborations for future projects. Researchers interested in accessing the model and the underlying image data for collaborative purposes can contact the corresponding author. The source code, including data preprocessing, the final model architecture, and model training pipeline is available at: https://doi.org/10.5281/zenodo.17097743.
